# Water requirement and growth indicators of forest tree species seedlings produced with automated irrigation management

**DOI:** 10.1371/journal.pone.0238677

**Published:** 2020-11-02

**Authors:** Mateus Marques Bueno, Paulo Sérgio dos Santos Leles, João Felício Gonçalves Abreu, Jaqueline Jesus Santana dos Santos, Daniel Fonseca de Carvalho

**Affiliations:** 1 Department of Agronomy, Federal Institute of Education, Science and Technology of Minas Gerais /IFMG, São João Evangelista, Minas Gerais, Brazil; 2 Department of Forestry, Forest Institute, Federal Rural University of Rio de Janeiro /UFRRJ, Seropédica, Rio de Janeiro, Brazil; 3 Department of Engineering, Institute of Technology, Federal Rural University of Rio de Janeiro /UFRRJ, Seropédica, Rio de Janeiro, Brazil; Hellenic Agricultural Organization - Demeter, GREECE

## Abstract

The lack of information regarding the water requirement of tree species results in water waste in the seedlings production in nurseries. Water requirement, the growth plant factors and water efficiencies for height and diameter were determined for *Schizolobium parahyba* (Vell.) Blake, *Cytharexylum myrianthum* Cham. and *Ceiba speciosa* Ravenna seedlings, under automated irrigation management and greenhouse conditions, located at 22^o^45’53” S and 43^o^41’50” W. We used sewage sludge biosolids as substrate in the seedling phase (280 cm^-3^ tube), and sandy soil material in the initial pot growth phase (18 dm^-3^ pot). In the seedlings phase, four water replacement levels were applied to the substrate, by drip irrigation, corresponding to average replacement ranging from 40 (V1) to 100% (V4) of the species water requirement. Seedlings developed properly and 80 days after emergence, *S*. *parahyba*, *C*. *myrianthum* and *C*. *speciosa* seedlings received, respectively, 2.40, 1.08 and 0.85 L per plant, for V4. After growth phase (230 DAE), the total water volumes were, respectively, 70.0, 50.3 and 52.7 L per plant. Under adequate water supply, there were rapid recovery and growth of the species, even for the seedlings which showed different height and diameter in the tube phase. The growth plant factors values found were below 0.5 for all species indicating low sensitivity to growth, both in height and in diameter, in response to water deficit. Water efficiency indicators point to distinct trends between the two phases, and *C*. *speciosa* has higher values of water efficiencies for height (80.7 and 17.0 cm L^-1^) and diameter (2.1 and 0.5 mm L^-1^) in both phases.

## Introduction

Areas requiring some sort of recovery in different degraded ecosystems of Brazil reach about 21 million hectares and are characterized as with legal deficit of native vegetation [1]. Much of this area is located in the Atlantic Forest biome, considered the most altered in Brazil, with an estimate of only 12.5% of its original cover [2], and one of the most important for conservation worldwide [3]. With the degradation defined in the context of a resilience-based approach, when core interactions and feedbacks are broken, human intervention is required to initiate a trajectory of recovery [4]. In this context, the planting of seedling has been one of the most used techniques for the Atlantic Forest restoration [5].

In the formation of stands aimed at forest restoration, it is essential to produce seedlings with high quality and rusticity, which are influenced by the availability of water, both over time and in terms of volume [6], and by the substrate used. Adequate supply of nutrients to seedlings can be performed by using sewage sludge biosolid as substrate [7], as it contains high organic matter content and has appreciable amounts of N and P [8].

Seedling quality is also related to the water supply in the nurseries, which should be carried out in response to the crop water requirement according to the interactions of the environment and water dynamics in the substrate [6]. About 49% to 72% of the water applied in reforestation nurseries is lost, varying according to the species [9]. In this context, drip irrigation associated with automated management can promot adequate water supply for the full development of the crop [10], using less water and labor [11].

Although there are reports on the growth of native tree species of the Atlantic Forest [12], most of them do not quantify the water requirement for seedlings, as well as their sensitivity to water deficit, which would contribute to the development of more efficient forest plantations. In order to represent the impact of the use of water resources in the tree seedlings production, we present news growth and water efficiency indicators respectively related to water deficit and water volume applied, for the development of seedlings in height and diameter.

The present study aimed to determine the water requirement, the growth plant factors (Gpf) and water efficiencies (WE) of Atlantic Forest tree species, under automated irrigation management in the seedling stage in plastic tube. Also, we evaluated the water requirement and WE during initial growth in pots, in a greenhouse.

## Material and methods

The experiments were conducted from September 2018 to May 2019 and consisted in the planting of seedlings of 3 species of native forest trees, *Schizolobium parahyba* (Vell.) Blake (Guapuruvu), *Cytharexylum myrianthum* Cham. (Pau Viola) and *Ceiba speciosa* Ravenna (Paineira), in 280 cm^3^ plastic tubes (phase 1) and in 18 dm^-3^ pots (phase 2).

The experimental areas are located in the Horticultural Sector of the Agronomy Institute of the Federal Rural University of Rio de Janeiro, municipality of Seropédica–RJ, Brazil (22°45'48" S, 43°41'50" W, altitude of 33 m). The regional climate classified by Köppen’s international system is Aw [13], humid climate and dry winter, with average annual rainfall of 1300 mm and average annual temperature of 22°C.

Seeds from mother trees of the Atlantic Forest of Rio de Janeiro were initially placed in sand boxes. After 20 days of emergence, when the seedlings were about 10 cm tall, their height and collar diameter were measured, and 60 homogeneous seedlings of each species were selected for transplantation to the tubes, which were installed in 4 trays, totaling 15 seedlings per tray. The material was placed on a metal workbench (0.8 x 1.2 x 3.0 m) and one micro-irrigation drip system was installed for each species, with independent flow rate sensors and independent reservoirs, located at 1.70 m above the workbench.

In the first phase, from 09/25/1918 to 11/24/2018, the experiments were conducted in a completely randomized design, with 4 treatments (irrigation levels) and 15 replicates (tubes per tray), in climate-controlled environment. The emitters used were spaghetti-type microtubing (Plasnova, mod. PDAEXT001000354), with 0.8 mm diameter and different lengths (0.80, 0.50, 0.35 and 0.20 m), promoting the different irrigation levels. After conducting flow rates tests (Table 1), distribution uniformity coefficients (DUC) greater than 95% were obtained.

**Table 1:** Mean flow rate of the emitters (L h^-1^) in the respective treatments, for the tree forest species.

Treatments	Experiments/Species		
	*S*. *parahyba*	*C*. *myrianthum*	*C*. *speciosa*
	Mean flow rate of the emitters (L h^-1^)		
V1	1.2	1.1	0.7
V2	1.8	1.5	1.1
V3	2.1	2.3	1.4
V4	2.9	2.6	1.8

The substrate used in the tubes was pure biosolid, obtained from a sewage treatment plant managed by the Rio de Janeiro State Water and Sewage Company (*Companhia Estadual de Águas e Esgotos*–CEDAE). Physical characteristics indicate the existence of large porous space (0.70 cm^3^ cm^-3^), low bulk density (0.74 g cm^-1^) and approximately 70% of the average particle diameter between 1.0 and 0.5 mm. The physical-hydraulic parameters of the substrate were obtained using the simplified evaporation method [14], through the commercial device Hyprop® [15], and indicated low water holding capacity. This substrate has sufficient levels of N (1.61%), P (0.68%), K (0.27%) and organic carbon (9.66%), which provide the mineral supply without the need for fertilization, especially in the initial growth stage of the seedlings.

Water management was performed by simplified irrigation controller (SIC) [16], which operates in response to soil water tension and is regulated by the level difference between a porous cap (sensor) and a pressure switch. This device has been used in several field studies [17] and greenhouse studies [18], using different soils or substrates for plants. It is presented as an alternative in irrigation management [19] and has the advantage of being built with low cost materials [20]. Two actuators were used for each plant species studied, independently installed in two tubes with treatment corresponding to the highest flow rate (V4), totaling 6 actuators for the 3 experiments.

The volume of water applied per treatment over time was measured by water flow sensors (mod. YF-S201b), connected to an Arduino MEGA board (mod. 2560), responsible for data storage. In order to avoid any percolation losses, a system composed of a rain sensor (mod) with direct actuation by relay was installed. Two sensors were installed in each experiment, just below the tube that received the largest volume of water.

At 60 days after the start of irrigation monitoring, when the seedlings reached average heights of 36.4 cm (*S*. *parahyba*), 25.9 cm (*C*. *myrianthum*) and 32.4 cm (*C*. *speciosa*), 16 seedlings of each species (4 of each treatment) were transferred to 18 dm^-3^ pots filled with soil material collected from the 0–40 cm layer of a *Quartzpsamments*, located in the municipality of Seropédica, RJ, Brazil. This soil had A horizon about 20 cm deep, followed by C horizon up to 120 cm. The dominant particle-size fractions were sand and loamy sand, the mean pH was 5.2 and base saturation (V) in the A horizon was equal to 36%, indicating low fertility.

Using the same experimental design, this second phase, totaling 48 seedlings, was conducted in a plastic greenhouse, simulating the field conditions, except for the water supply, performed by irrigation. In a period of 150 days, from 11/24/2018 to 04/24/2019, a uniform water depth was applied for all plants of each species by the independent irrigation system whose management was also carried out by the AAI, with sensors installed in the seedlings of V4 treatment. The irrigation system consisted of one dripper per pot (mod. PCJ—Netafim), with nominal flow rate of 2.0 L h^-1^, and the water volume applied was measured by previously calibrated flowmeter (Alpha mnf/FAE) installed in the main line. Uniformity tests indicated DUC above 97.0%. At 150 days after emergence, *C*. *myrianthum* plants required fertilization, because they had visual symptoms of nutritional deficiency.

The meteorological monitoring of the experiments was carried out by a WatchDog 2000 Series weather station (Spectrum Technologies, Illinois/USA), containing sensors of temperature, relative humidity, solar radiation and wind speed, and with data storage every 30 minutes. The average daily temperature during the first phase was 26.6 ^o^C, with little variation due to the climate-controlled environment. The average relative humidity was around 80% and the average solar radiation was 8.1 MJ m^-2^ day^-1^. In second phase, the average daily temperature was 27.4°C, with average amplitude of 10.8°C. The average relative humidity was around 44.0% and the average total radiation was 21.0 MJ m^-2^ d^-1^.

In both phases, nondestructive growth analyses for plant height (cm, distance from plant collar to apical bud, with a graduated ruler) and collar diameter (mm, with a digital caliper) were performed every 15 days, in first phase, and every 30 days, in second phase.

To verify whether the premises of the analysis of variance were met, the normality and homogeneity of the residuals were assessed by the Bartlett *test*, at the level of 5% probability. When these criteria were met, the analysis of variance was performed using the t test, with a significance level of 5%. After rejecting the null hypothesis, polynomial regression analyses were performed for the accumulated volume applied and irrigation regime factors. All statistical analyses in this study were performed using software package R, version 3.6.0.

Using the same concepts of the yield response factor (Ky) [21], the growth plant factor related to water deficit for the development of seedlings in height and diameter (Gpf) was calculated by Eq 1.

$$\left(1-\frac{V_{a}}{V_{m}}\right)=Gpf\;\left(1\frac{{Vol}_{a}}{{Vol}_{m}}\right)$$ (1)

where V_a_ is actual value of the variable (height—cm, or diameter—mm), V_m_ is maximum value of the variable (height—cm, or diameter—mm), Vol_a_ is actual volume applied (L) and Vol_m_ is maximum volume applied (L).

Since this relationship above is linear, Gpf corresponds to the slope of the regression line, which was obtained iteratively by maximizing the V_m_ value (height or diameter) so that the equation intercept with the ordinate axis became equal to zero. The procedure was performed in an electronic datasheet (MS Excel^TM^), using the Solver module [22]. The interpretation of Gpf was done according to [23], for Ky.

Water efficiency (WE) based on height (HWE) and diameter (DWE) was obtained according to Eq 2.

$$WE=\frac{Vg}{Tva}$$ (2)

where Vg is the variable growth (height—cm, and diameter—mm) and Tva is the total volume applied, in L per plant.

## Results and discussion

At 60 days after the start of irrigation monitoring, from 20 to 80 days after emergence (first phase), the species reached on average the minimum standard for field planting in all treatments, which corresponds to the range from 20 to 40 cm for height and diameter greater than 3 mm [24], for quality seedlings produced in 280 cm^3^ tubes. During this period, the irrigation systems were actuated 73, 43 and 52 times for the *S*. *parahyba*, *C*. *myrianthum* and *C*. *speciosa* species, respectively, applying total volumes of 0.91, 1.49, 1.74 and 2.40 L per plant, 0.46, 0.62, 0.95 and 1.08 L per plant, and 0.33, 0.52, 0.66 and 0.85 L per plant, for V1, V2, V3 and V4, respectively. The irrigation system was turned on more than once a day for the three species in 14, 5 and 10 days (2 activations), and in 6, 3 and 4 days (3 activations), respectively, for the *S*. *parahyba*, *C*. *myrianthum* and *C*. *speciosa* species. In addition to meeting the seedlings water requirements, the large number of actuations of the SIC is associated with the low water holding capacity in the substrate.

**Fig 1:**
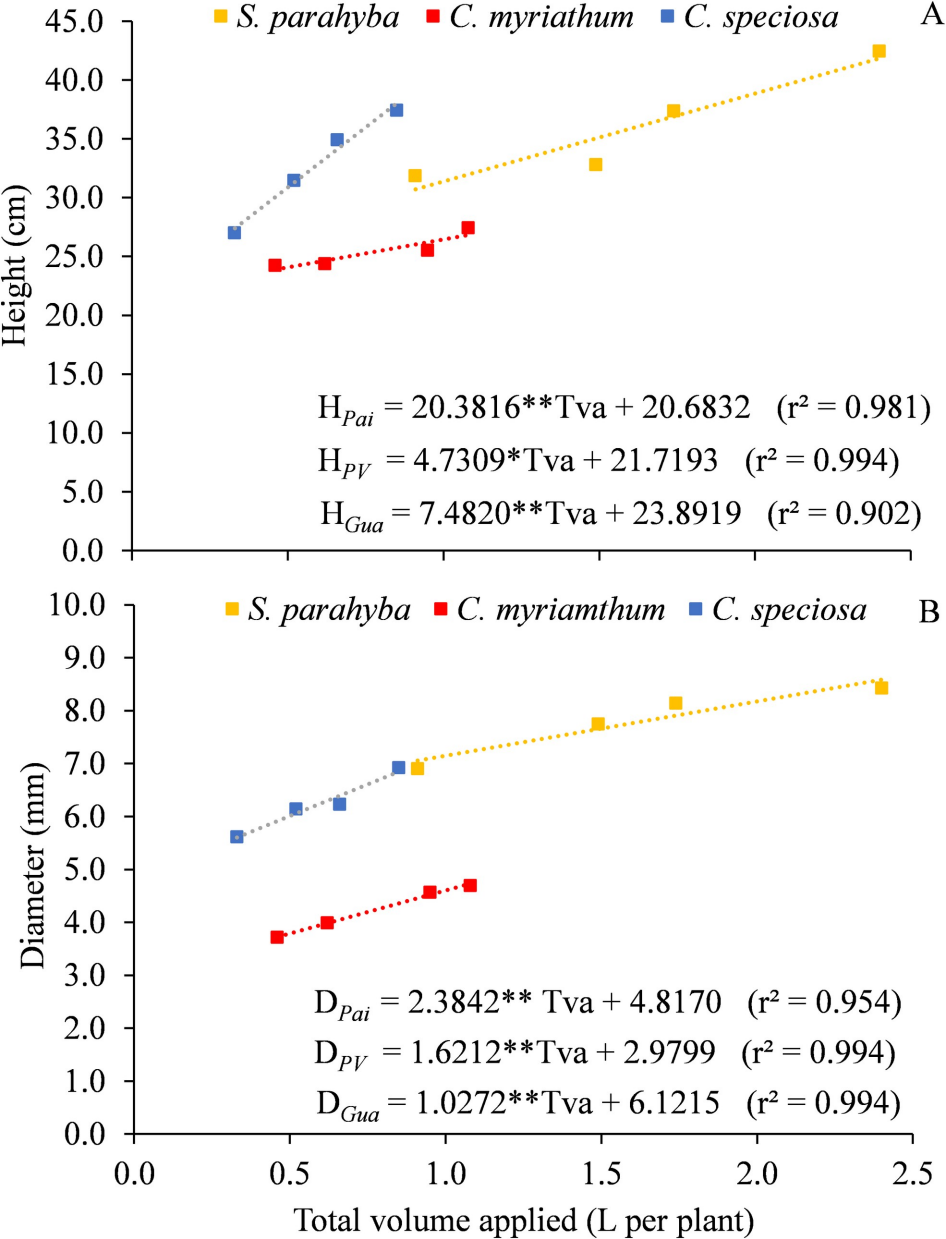
Variations of height (A) and diameter (B) as a function of the total volume applied (Tva) on treatments for three forest species, in the tube phase. *p<0.05, **p<0.01.

There were positive responses of height (H) and collar diameter (D) as a function of the increase in water volumes applied to the seedlings of all species (Fig 1).

It is worth pointing out that the SIC used in this study provides water for plants in response to their development [16], maintaining the moisture corresponding to the water capacity of the substrate. Thus, the volumes of 2.40, 1.08 and 0.85 L per plant, for the V4 treatment, correspond to the water requirement for the *S*. *parahyba*, *C*. *myrianthum* and *C*. *speciosa* species, respectively.

The variations in height (Fig 1A) and diameter (Fig 1B) of the species were proportional to the different volumes applied, justifying the linear fit of the regression models. The lowest angular coefficients indicate lower responses to seedling growth and were obtained for *C*. *myrianthum* (4.7309 cm L^-1^ –height) and *S*. *parahyba* (1.6212 mm L^-1^ –diameter). *C*. *speciosa* species had the highest growth rates in response to the amount of water applied, with values of 20.3816 cm L^-1^ in height (Fig 1A) and 2.3842 mm L^-1^ in diameter (Fig 1B). The amount of water required by *S*. *parahyba* seedlings was, on average, 171.1% higher than the volume applied to the *C*. *speciosa* seedlings, although the differences in height (10.7%) and diameter (25.4%) were proportionally smaller. The difference in the relative water demand for these two species probably occurred due to their phytoecological regions of occurrence. According to [25], *S*. *parahyba* occurs in the alluvial plain and beginning of the slopes, while *C*. *speciosa* frequently occurs in different environments, such as in some areas of the Caatinga domain. *C*. *myrianthum* had the lowest growth in height and diameter, reaching just 27.43 cm and 4.69 mm, respectively. As it is a non-pioneer species, its lower growth is explained by the lack of shading precisely in the initial stages of development [26].

Calculated from Fig 1, the maximum height/diameter ratio (H/D) values for *S*. *parahyba* (5.0), *C*. *speciosa* (6.5) and *C*. *myrianthum*. (5.6) are within the range below 8.0 indicated by [24]. This ratio is used to assess the quality of forest seedlings, because, in addition to reflecting the accumulation of reserves, it ensures greater resistance and better fixation in the soil [27]. The occurrence of water deficit in the substrates tends to limit the growth of tree seedlings in terms of diameter and height and modify the ideal H/D ratio for a given species [28]. However, the variations in the H/D ratio observed in this study were not enough to provide signs of etiolation in the seedlings. According to [29], the effects of irrigation on the height and diameter of tree or fruit species seedlings vary depending on the plant, size, growth habit, leaf type and size, substrate characteristics and cultivation container, besides the microclimatic conditions of the growing environment.

**Fig 2:**
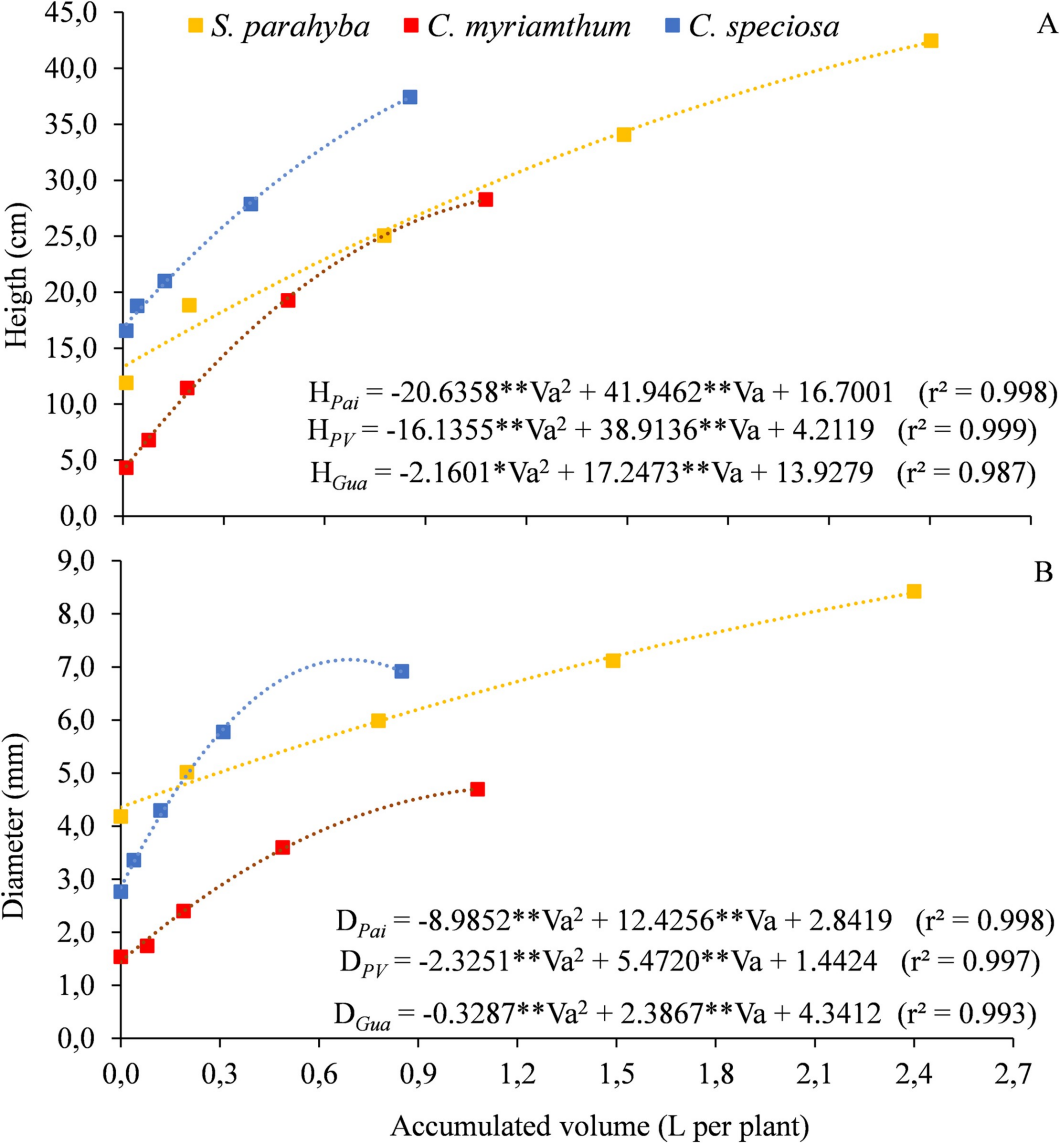
Variation of height (A) and diameter (B) as a function of the water volume applied throughout the experiment (Va) for three forest species, in the treatment with 100% water replacement (V4) for the tube phase. *p<0.05, **p<0.01.

The growth of the seedlings in relation to the water volumes applied per plant throughout the first phase in the V4 treatment is presented in terms of height (Fig 2A) and diameter (Fig 2B) for the three species. *S*. *parahyba* reached greater height (42.5 cm) and diameter (8.4 mm), followed by *C*. *speciosa* (37.4 cm, 6.9 mm) and *C*. *myrianthum* (27.4 cm, 4.7 mm). Using a substrate composed of organic matter, peat and vermiculite, under field conditions, *S*. *parahyba* seedlings reached a maximum height of 36.8 cm, with daily wetting carried out manually [30].

In the regression models, the low coefficients of the quadratic terms for *S*. *parahyba* mean a trend of linearity in the growth of seedlings in height (Fig 2A) and diameter (Fig 2B), especially after the second analysis, performed on 10/10/2018, indicating a more uniform development throughout the evaluation period. *C*. *myrianthum* started the development phase in tubes with the smallest height (4.3 cm) and diameter (1.5 mm) and showed an intermediate growth rate. The highest growth rate in height (Fig 2A) and diameter (Fig 2B) was found for *C*. *speciosa*, despite the lower total water volume applied (0.85 L per plant).

The growth of the species in terms of height and diameter increased proportionally with the dates of evaluation, consequently demanding greater volumes of water. From the first to the second (09/25 to 10/10) and from the fourth to the fifth growth analysis (11/09 to 11/24), the seedlings of *S*. *parahyba*, *C*. *myrianthum* and *C*. *speciosa* grew, respectively, 6.9 and 8.4 cm, 2.5 and 8.1 cm, and 2.2 and 9.6 cm in height (Fig 2A). In terms of diameter, the variations were 0.8 and 1.3 mm, 0.2 and 1.1 mm, and 0.6 and 1.1 mm, respectively (Fig 2B). In these periods, the applied volumes were 0.02 and 0.91 L per plant, 0.08 and 0.59 L per plant, and 0.04 and 0.54 L per plant, respectively. In addition, from 09/11 to 11/24, the SIC was turned on 31 (42.5%), 24 (55.8%) and 29 (55.8%) times for the *S*. *parahyba*, *C*. *myrianthum* and *C*. *speciosa* species, respectively, applying 0.910 (37.9%), 0.5907 (54.6%) and 0.540 (63.5%) L per plant. These values indicate the importance of an adequate irrigation management in order to meet the seedlings water requirements, which vary over time.

Under pot conditions, simulating cultivation in the field, the water volumes applied were 70.0, 50.3 and 52.7 L per plant, respectively, for the *S*. *parahyba*, *C*. *myrianthum* and *C*. *speciosa* species, with 182, 150 and 171 actuations of the irrigation systems. The irrigation systems were turned on more than once a day in 29, 18 and 29 days (2 activations), and in 28, 22 and 24 days (3 activations), respectively, for the *S*. *parahyba*, *C*. *myrianthum* and *C*. *speciosa* species.

The heights and diameters of the species did not differ statistically at 5% significance level, by Tukey test, over the 5 months of evaluation, indicating that under the condition of adequate water supply there is rapid recovery and growth of the species, even for those which showed different diameter and height in the tube phase (Fig 1). Thus, through the automatic management of the irrigation system by SIC, the supply of water to plants was adequate and guaranteed full development of the seedlings. However, under field conditions, this situation may not occur due to increases in temperature and the intensity and frequency of drought periods caused by climate change [31].

**Fig 3:**
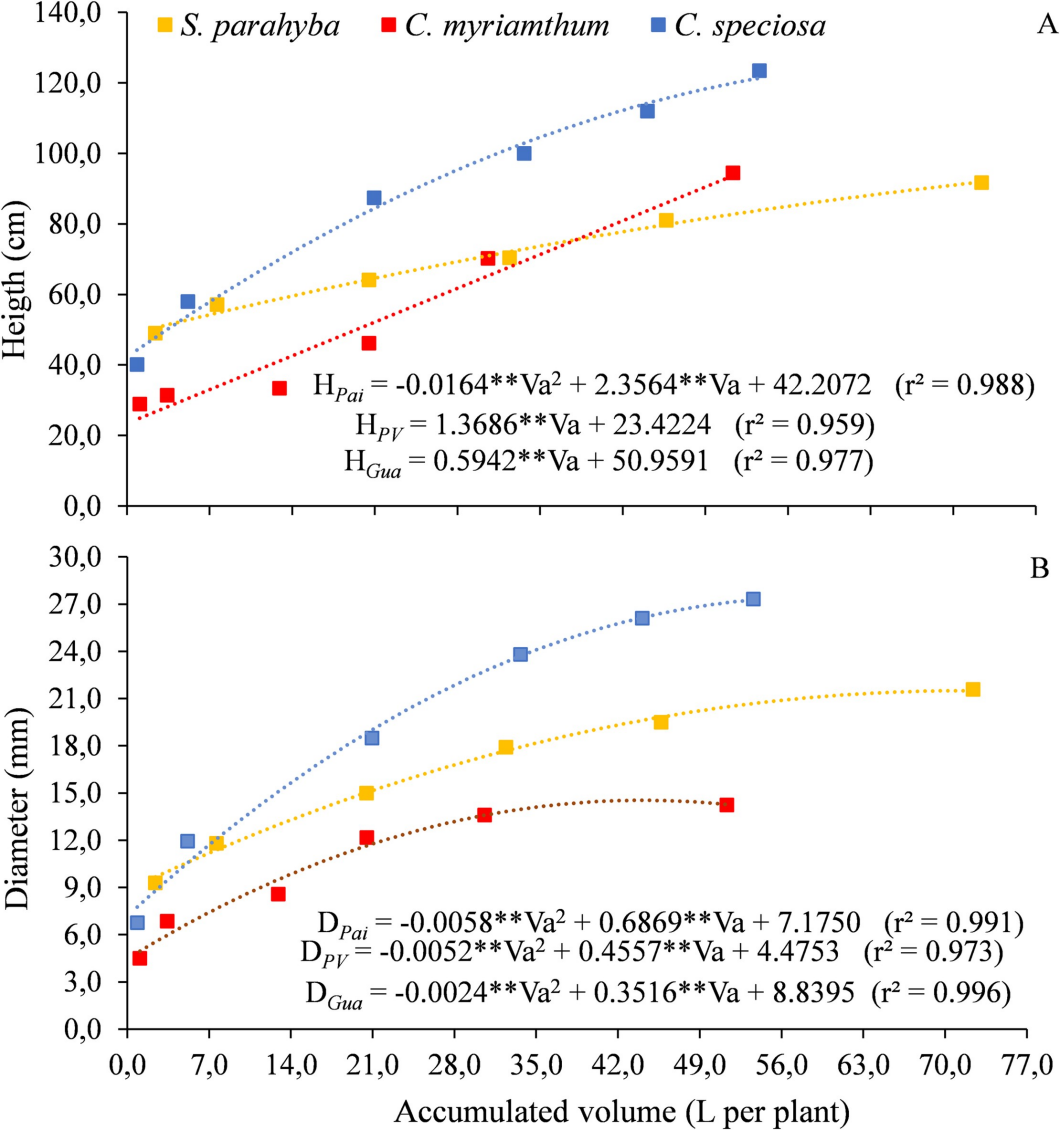
Variation of height (A) and diameter (B) as a function of the water volume applied throughout the experiment (Va) for three forest species, in the pots phase. **p<0.01.

The growth of the seedlings in terms of height (Fig 3A) and diameter (Fig 3B) indicate a greater water use for *S*. *parahyba*, but *C*. *speciosa* reached greater height (123.5 cm) and diameter (27.3 mm). The amount of water applied to *C*. *myrianthum* species was similar to that applied to *C*. *speciosa*, although the seedlings reached, on average, 94.5 cm in height and 14.2 mm in diameter.

The *S*. *parahyba* species showed the same trend observed in the tube growth phase, with a lower rate compared to the other species and a uniform growth between the evaluation dates (Fig 3A). From the first to the second (11/24/2018 to 12/24/2018) and from the fifth to sixth growth analysis (03/23/2019 to 04/24/2019), the seedlings grew 8.1 and 10.8 cm, respectively, with applied volumes of 5.24 and 26.7 L per plant. In these periods, the average height and volume applied to *C*. *myrianthum* seedlings varied, respectively, between 2.5 and 24.3 cm, and between 2.3 and 20.8 L per plant.

These species showed a linear trend of growth in height, while *C*. *speciosa* showed a second-order polynomial trendy, despite the low coefficient of the quadratic term (-0.0164) (Fig 3A). Unlike the previous ones, *C*. *speciosa* seedlings had higher growth in height (29.38 cm) and applied volume (15.78 L per plant) between the second (12/24/2018) and third (01/23/2019) measurements, when the irrigation system was turned on 51 times. In this period, the highest incidence of radiation (24.7 MJ m^-2^ d^-1^) and the maximum temperature (40.4 ^o^C) of the experiment were recorded.

The greater volume of water applied to *S*. *parahyba* from the fifth to sixth growth analysis (26.7 L per plant) was sufficient to promote 2.10 mm growth in diameter, while for *C*. *myrianthum*, the volume of 20.75 L per plant promoted variation in diameter of just 0.6 mm, in the same period (Fig 3B). For these species, the irrigation systems turned on 54 and 43 times, respectively. The volume of 15.78 L per seedling of *C*. *speciosa*, applied between the second and third measurements led to a variation in the diameter of 6.6 mm. There was greater proportional growth in diameter than in height for the species *S*. *parahyba* and *C*. *speciosa*, while for *C*. *myrianthum* this growth was similar.

[26] conducted a comparative study between pioneer and non-pioneer species of the Atlantic Forest and concluded that *C*. *speciosa* and *S*. *parahyba* are among those with the highest growth rates, receiving scores of 8 and 9, respectively, in a classification from 0 to 10, to compose projects of recovery of degraded areas. [32] evaluated native forest species in mixed plantations in Brazilian Savanna at 6.4 years old and *C*. *speciosa* was among those with the highest growth, having average stocks of volume and biomass of 114.03 m^3^ ha^-1^ and 52.99 Mg ha^-1^, respectively.

*S*. *parahyba* is a fast-growing tree (up to 45 m^3^ ha^-1^ yr^-1^) recommended and widely cultivated as ornamental or for restoration purposes [33], and not being very demanding in terms of soil fertility, which facilitates its adaptation in different locations [26]. For 13 years, [34] evaluated the development of an area of regeneration with *S*. *parahyba* in the Amazon region. The species showed a volume increase of 3.1 m^3^ ha^-1^ year^-1^ for individuals with diameter greater than 25 cm and more than 30% of the planted seeds were able to germinate, establish and grow until reaching a diameter greater than 25 cm. This field result combined with the present study indicates that *S*. *parahyba* has high capacity for water use and, consequently, for forming stands.

**Fig 4:**
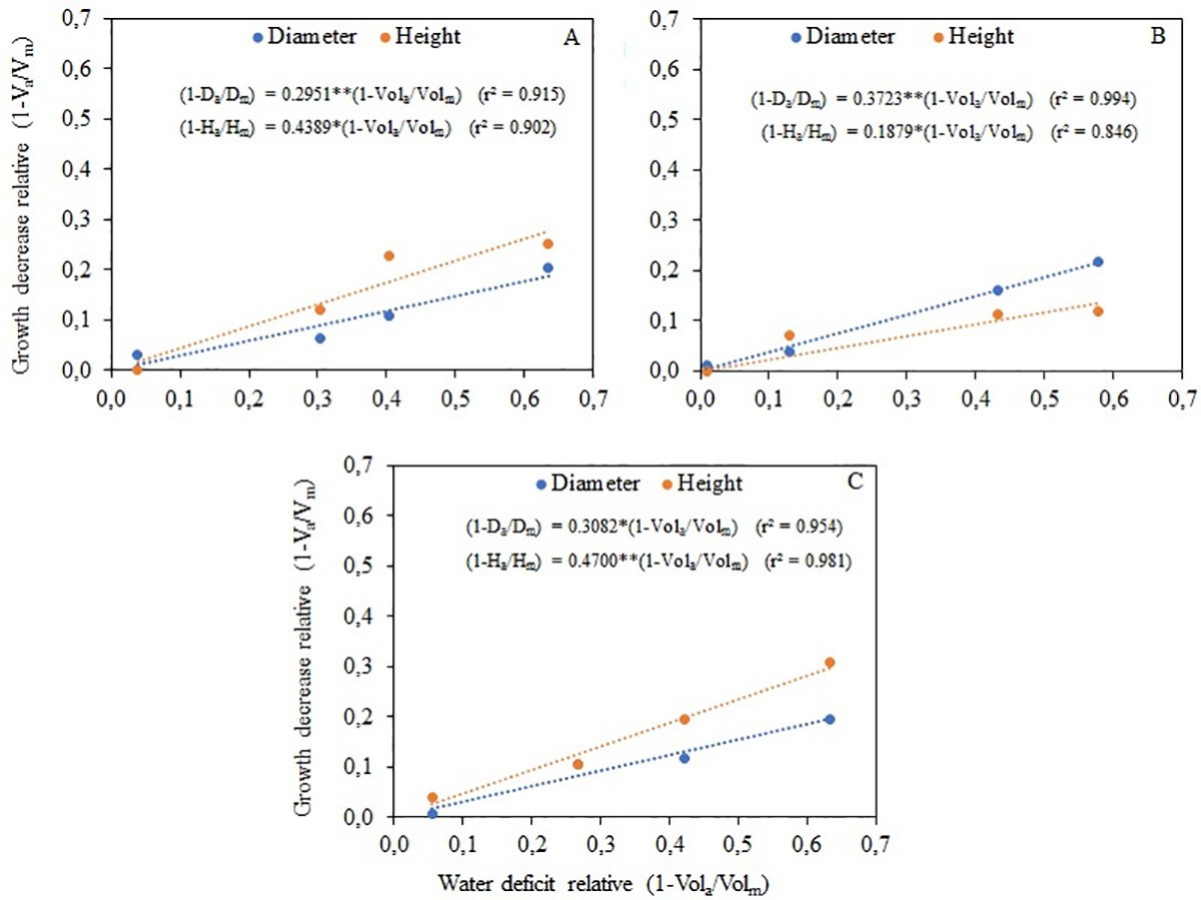
Relationship between decrease relative of growth, in height and diameter, and water deficit for *S*. *parahyba* (A), *C*. *myrianthum* (B) and *C*. *speciosa* (C) seedlings. *p<0.05, **p<0.01.

The Gpf values found were below 0.5 for all species, as a function of both height and diameter (Fig 4), indicating low sensitivity to water deficit, despite showing rapid growth.

This trend is confirmed by the results obtained in phase 2 (pots) when seedlings of all species from the different treatments in phase 1 (tubes) had satisfactory development, showing rapid recovery and growth under adequate water supply. The lowest and highest Gpf values (Fig 4) obtained refer to the height variable for the *C*. *myrianthum* (0.1879) and *C*. *speciosa* (0.47) species, and they are related to the respective slopes observed in the response functions of Fig 1A.

We understand that the interpretation of the proposed index (Gpf) helps in the management of seedling production systems, however it cannot be compared numerically with other results in the literature as it is not normally used to evaluate the sensitivity of forest seedlings to water deficit. In any case, [6] state that the low sensitivity of forest tree species to water deficit in the seedling phase occurs because they have only one vegetative stage. It can be affirmed that the reductions in height and diameter are relatively small compared to the evaluated levels of water deficit, indicating that the species have high capacity for forming stands in initial processes of regeneration, even under conditions of low rainfall, provided that there is minimal water supply in the soil.

Water efficiency (WE) indicators point to distinct trends between the two phases (tube and pot), when the values are compared as a function of height (HWE) and diameter (DWE) (Table 2). In general, WE increases with the reduction in the volume applied for tube phase, and higher values are obtained for *C*. *speciosa*, followed by *C*. *myrianthum* and *S*. *parahyba*. In the pot phase, the differences between the values are reduced due to the recovery observed in seedlings from the treatments with deficits applied in phase 1. Except for *S*. *parahyba* in treatment V4, all HWE values obtained are statistically equal for the respective species.

**Table 2:** Water efficiency indicators related to total volume applied for the development of seedlings in height (HWE) and diameter (DWE), for *S*. *parahyba*, *C*. *myrianthum* and *C*. *speciosa* species.

Species	Treatment	Water efficiency indicators			
		HWE (cm L^-1^)		DWE (mm L^-1^)	
		tube	pot	tube	pot
*S*. *parahyba*	V1	35.018 a*	1.072 ab	7.582 a	0.296 a
	V2	22.013 b	0.986 b	5.196 b	0.297 a
	V3	21.475 cd	0.993 b	4.676 bc	0.283 a
	V4	17.694 d	1.267 a	3.510 c	0.298 a
*C*. *myrianthum*	V1	52.676 a	1.760 a	8.074 a	0.257 a
	V2	39.343 b	2.010 a	6.431 b	0.300 a
	V3	26.877 c	1.909 a	4.808 c	0.318 a
	V4	25.401 c	1.841 a	4.345 c	0.277 a
*C*. *speciosa*	V1	80.653 a	2.055 a	17,002 a	0.469 a
	V2	60.513 b	2.052 a	11.809 b	0.477 a
	V3	52.930 bc	2.098 a	9.433 c	0.482 a
	V4	44.039 c	2.305 a	8.138 d	0.510 a

*Means followed by the same letter in the column do not differ by Tukey test at 5% probability level, for the same species.

Although the best seedlings development was observed under 100% water suppression, the field monitoring and water efficiency values showed that there may be a good development even in seedlings that received less water. In absolute terms, *C*. *speciosa* was the species that had the highest values of water efficiency, indicating greater capacity to use this resource. It must be pointed out, however, that all species showed satisfactory growth and were efficient in the use of water, thus being recommended for processes of regeneration of native forests. Tree planting contributes to a rapid recovery of the forest structure, thus providing an adequate habitat for the restoration of the ecological succession [35].

## Conclusions

The water requirement for forest species is not normally known by nursery managers, as well as the effect of water stress on the seedling production stage. Despite the importance of saving water in production systems, this topic is understudied and there is no much information about the amount of water needed to produce quality seedlings in Brazil and worldwide.

We evaluated the response of three forest species under different water replacement levels when planted in substrates composed of pure biosolid. *S*. *parahyba*, *C*. *myrianthum* and *C*. *speciosa* species exhibit greater growth when subjected to automated irrigation management, at a level of 100% water supply. The total volumes of 2.40, 1.08, and 0.85 L per plant (Fig 1), respectively, for *S*. *parahyba*, *C*. *myrianthum* and *C*. *speciosa* species, can be used as a reference for commercial nursery irrigation projects, under the same environmental conditions as the experiment.

Growth plant factors (Gpf) for the seedling stage are lower than 0.5 (Fig 4), indicating low sensitivity to growth in the face of water deficit.

The best level of water efficiency was found for *C*. *speciosa*, indicating that this specie has greater capacity to use this resource (Table 1).

The studied species show rapid growth and are efficient in the water use, which implies a good development under conditions of variable water availability, common in the reforestation processes.
